# Supplementary material for: Water Parameters and Hydrodynamics in Rivers and Caves Hosting Astyanax mexicanus Populations Reveal Macro‐, Meso‐ and Microhabitat Characteristics
Source: Ecol Evol. 2026 Jan 25;16(1):e72970. doi: 10.1002/ece3.72970 (PMC12832201; doi:10.1002/ece3.72970)

Supplemental file 1

Maps of caves studied and sampling locations

Cueva de El Pachón

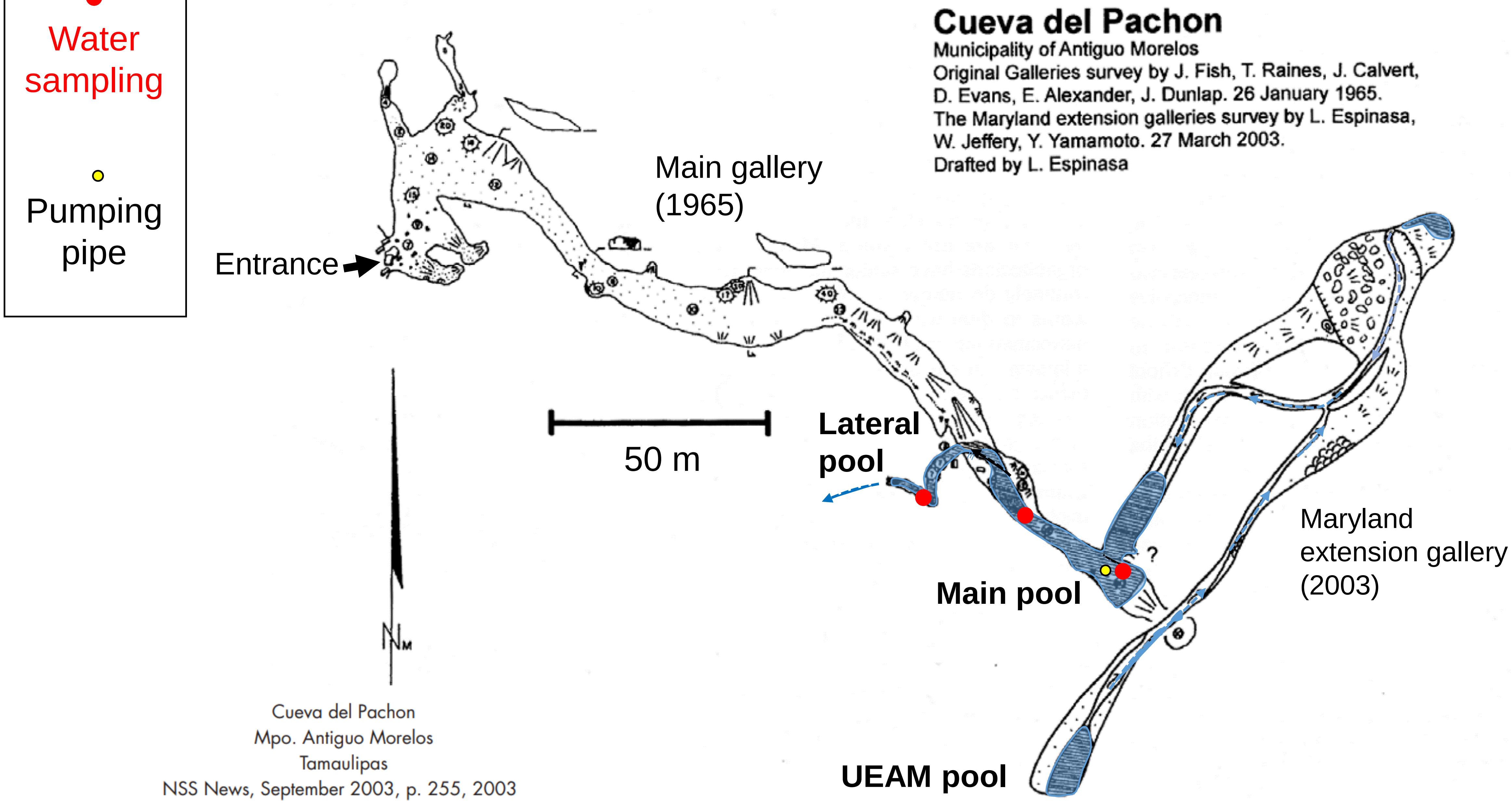

Cueva del Pachon
Mpo. Antiguo Morelos
Tamaulipas
NSS News, September 2003, p. 255, 2003
Astyanax International Meeting 2009 program and abstracts, p. 16, 2009

● Water sampling

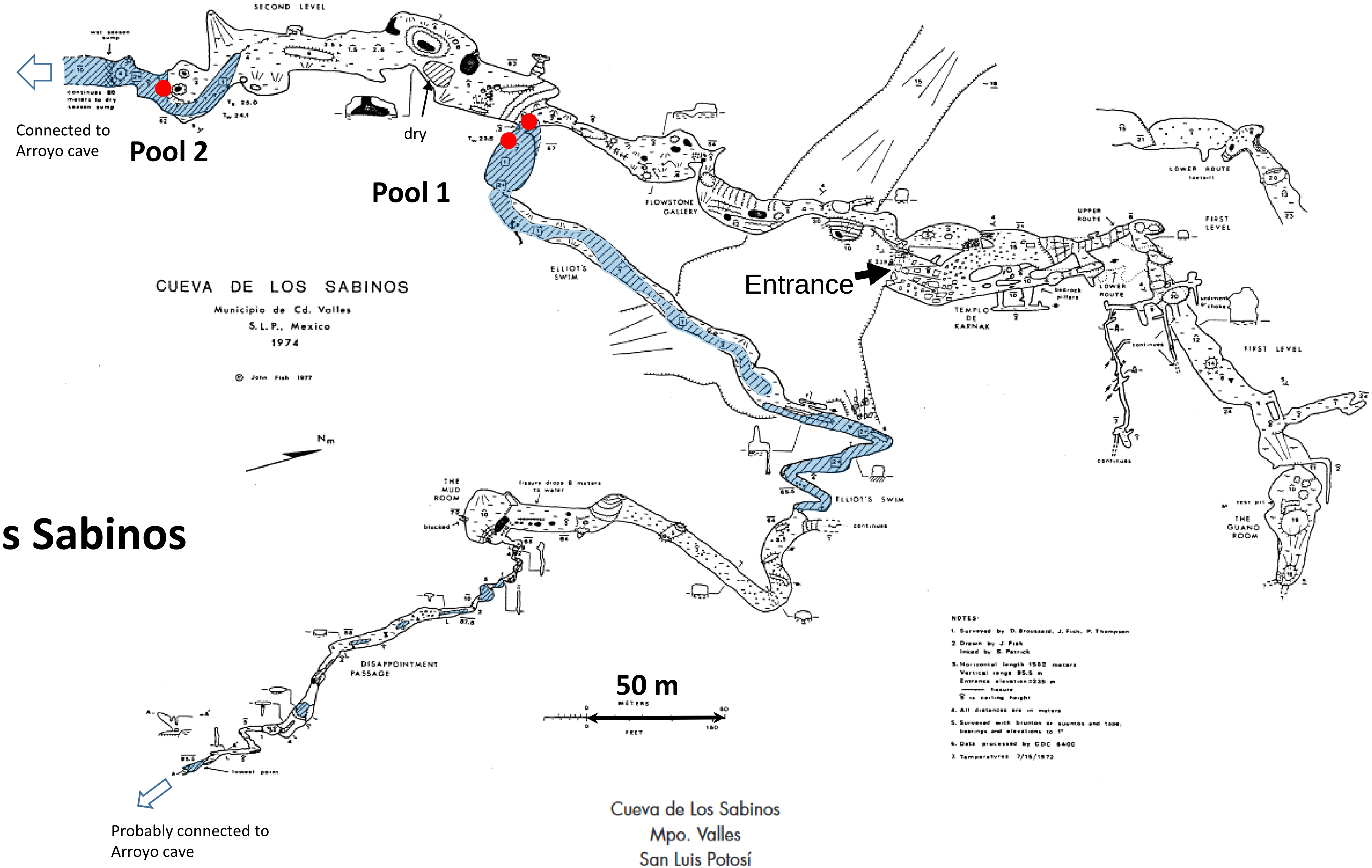

Cueva de Los Sabinos

Cueva de Los Sabinos
Mpo. Valles
San Luis Potosí
Karst Hydrology of the Sierra de El Ebra, Mexico, p. 117
Canadian Caver, v. 6, no. 1, p.15, 1974

Sótano de las Piedras

Water sampling

Brunton compass & tape surveys by William R. Elliott, Jim McIntire & Don Broussard, 17 July 1969; William R. Elliott, Robert Hemperly, John Prentice & Carmen Soileau, 19-20 May 1974. Entrance elevation 145 m, depth 52 m, horizontal length 434 m, extent 468 m. Redrawn by Elliott, 2014.

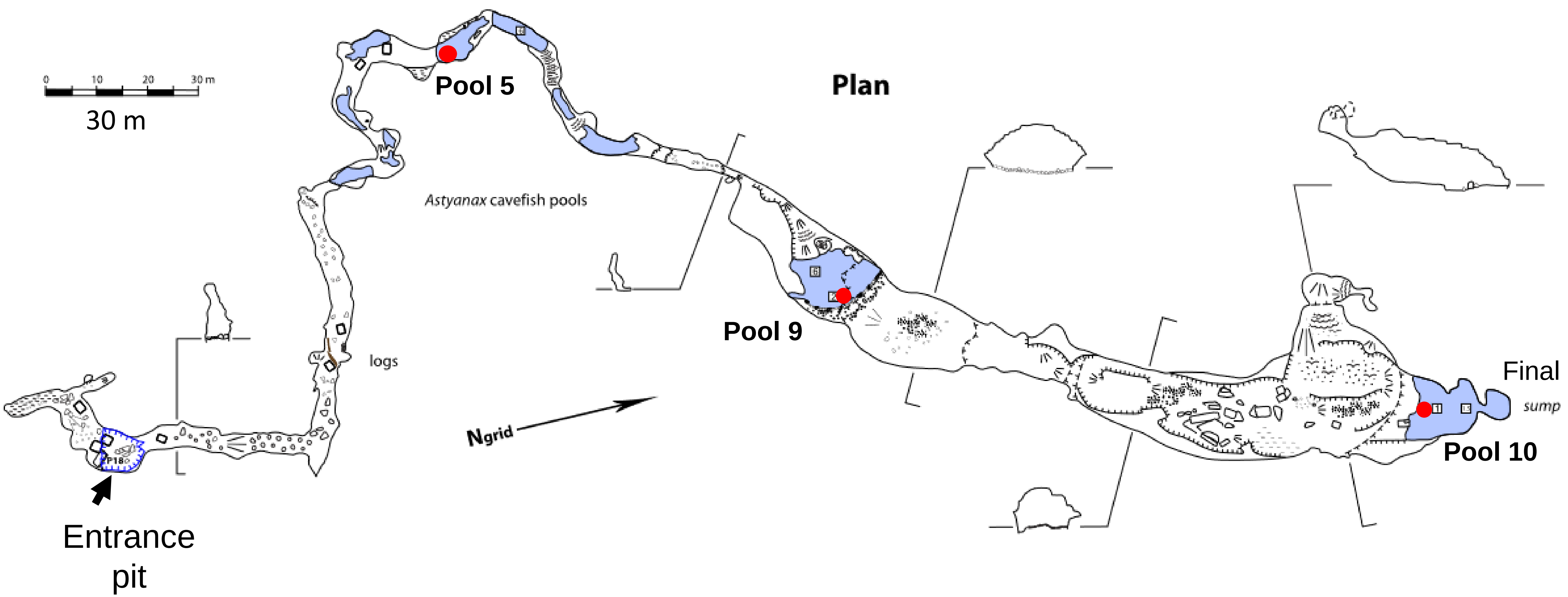

Water sampling

Sótano de la Tinaja

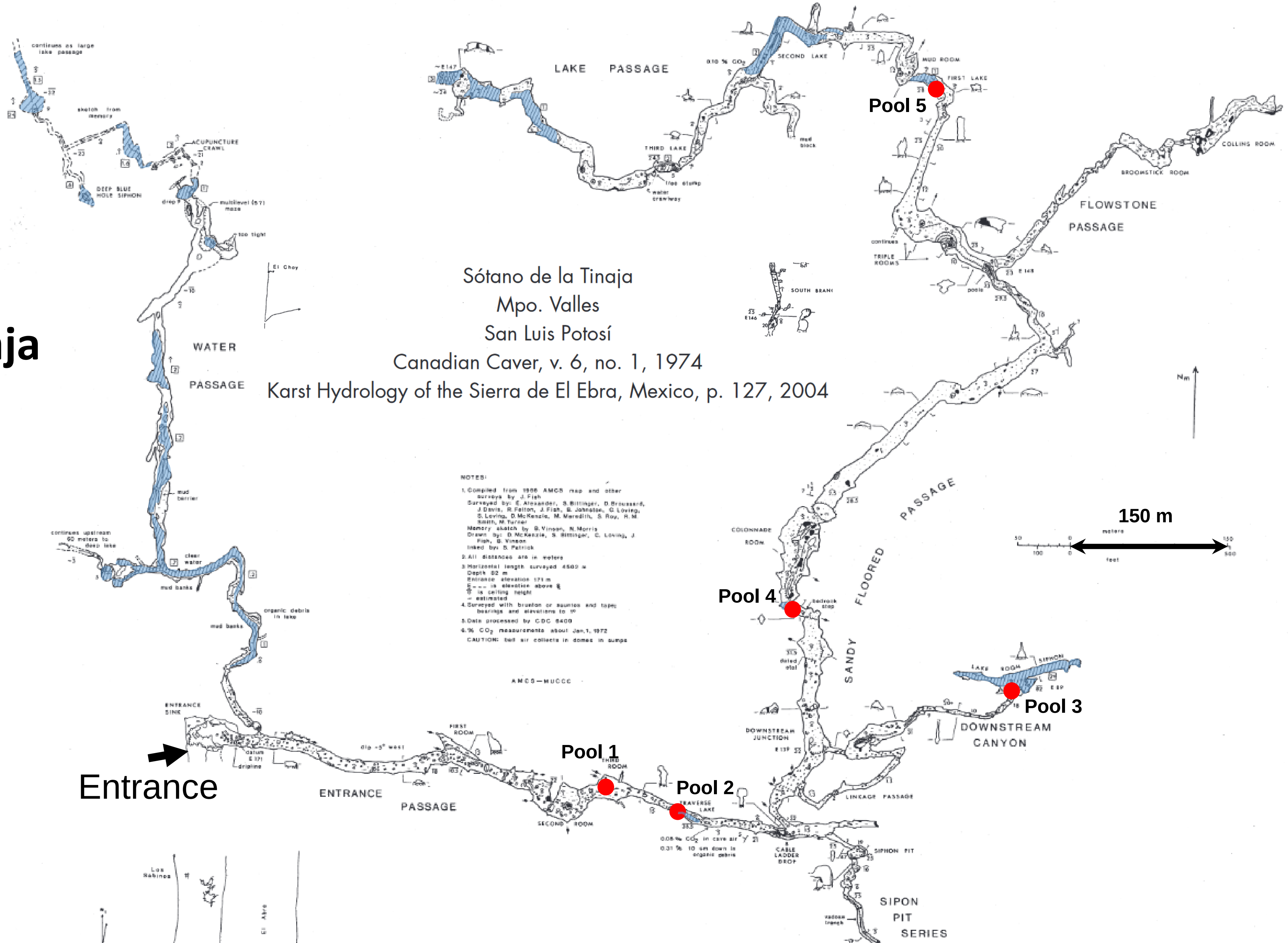

Cueva Chica

● Water sampling

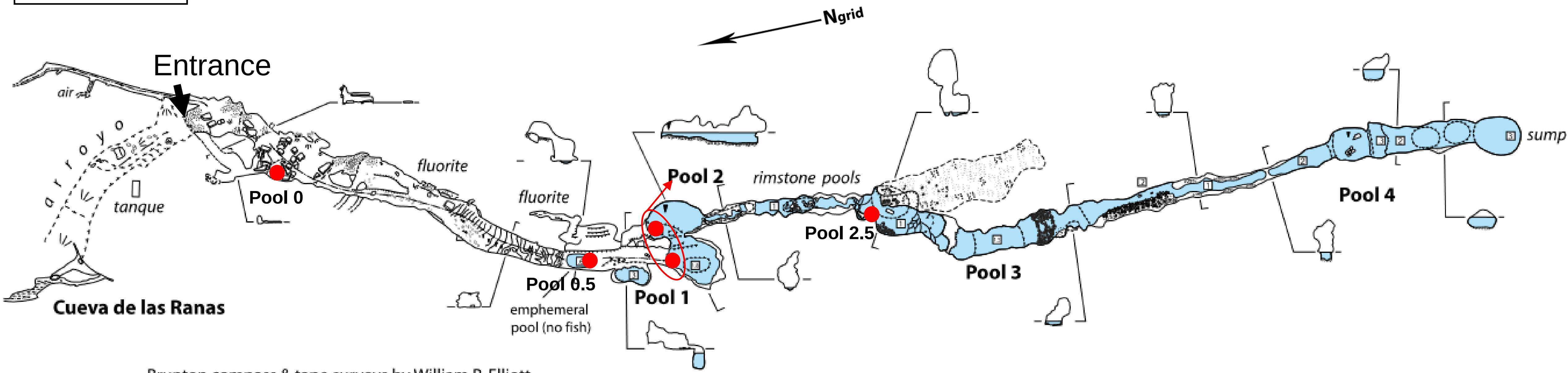

Brunton compass & tape surveys by William R. Elliott,
Bill Russell, Russell Harmon, Mel Brownfield, Suzanne Wiley &
Carmen Soileau. 8 Jan. 1970, 20 March 1971, 21 May 1971,
23 May 1971, 22 May 1974. Redrawn by Elliott, 2014.
Entrance elevation ~68 m, depth 19 m, relief 27 m, horizontal length
573 m, extent 591 m. Entrance to sump 280 m at 192° from grid north.

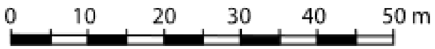

Cueva del Río Subterráneo

Water sampling

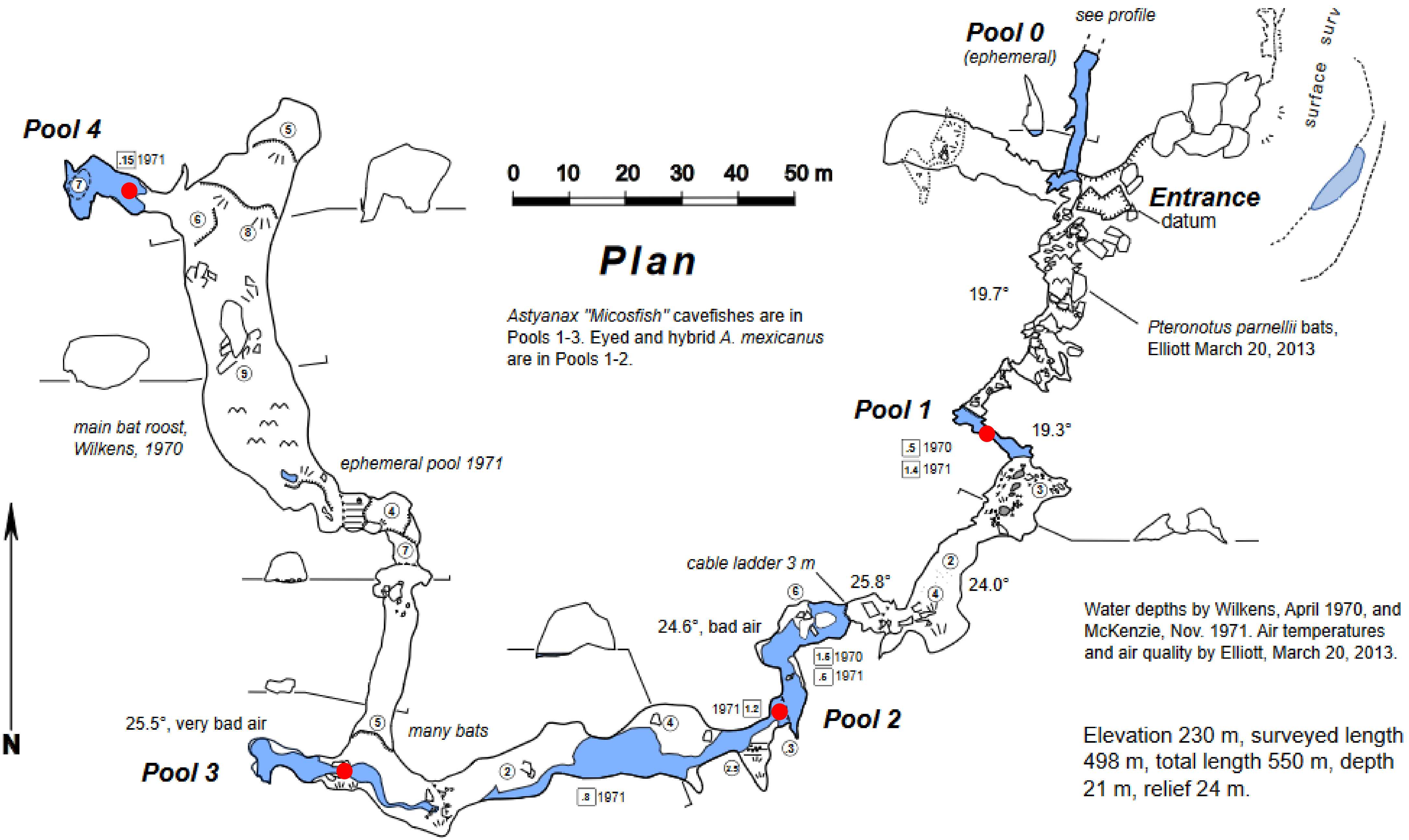

Supplement: Supplementary file 1 — Appendix S1: Supporting Information. [file ECE3-16-e72970-s002.pdf]
