# Supplementary material for: Water Parameters and Hydrodynamics in Rivers and Caves Hosting Astyanax mexicanus Populations Reveal Macro‐, Meso‐ and Microhabitat Characteristics
Source: Ecol Evol. 2026 Jan 25;16(1):e72970. doi: 10.1002/ece3.72970 (PMC12832201; doi:10.1002/ece3.72970)
Supplement: Supplementary file 2 — Appendix S2: Supporting Information. [file ECE3-16-e72970-s001.pdf]

Supplemental Information Appendix

Water parameters and hydrodynamics in rivers and caves hosting *Astyanax mexicanus* populations reveal macro-, meso- and micro-habitat characteristics

Laurent Legendre[1]*, Stéphane Père[2], François Rebaudo[1], Luis Espinasa[3], Joël Attia[4], Sylvie Rétaux[2]*

[1] EGCE UMR9191, CNRS, IRD, University Paris-Saclay, 91190 Gif-sur-Yvette, France

[2] Paris-Saclay Institute of Neuroscience, UMR9197 CNRS and University Paris-Saclay, 91400 Saclay, France

[3] School of Science, Marist College, Poughkeepsie, New York 12601, USA

[4] Equipe de Neuro-Ethologie Sensorielle, CRNL, CNRS and Université de St Etienne, France

* Authors for correspondence:
laurent.legendre@universite-paris-saclay.fr & laurent.legendre@cnrs.fr
sylvie.retaux@cnrs.fr

Running title: The hydrology of *Astyanax mexicanus* cavefish habitat

Key words: underground water, cavefish biology, cave environment, ecology, evolution

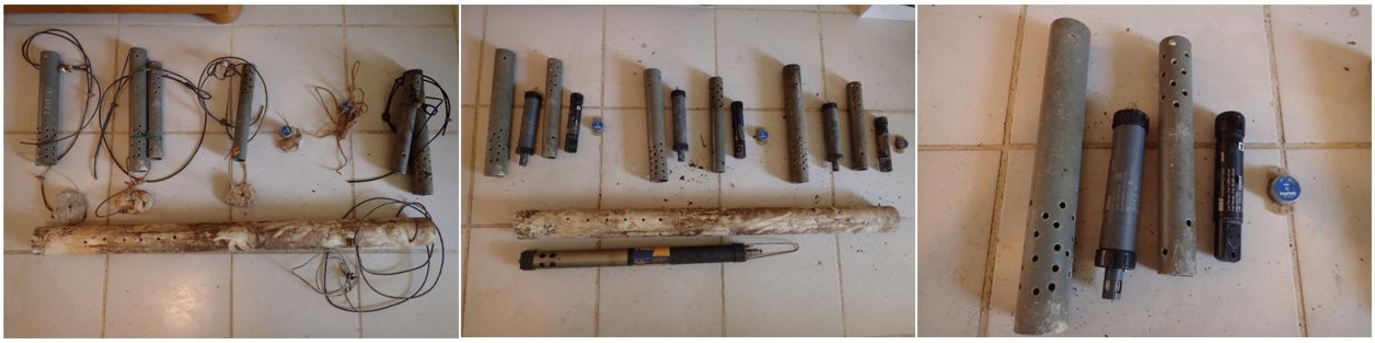

Supplemental Figure 1: Equipment used.

Photographs illustrating the state of conservation of probes (VuSitu Aquatroll600 and Hobo probes) and their fixation and protection systems, after one year spent in the natural cave environment. Photos by LL.

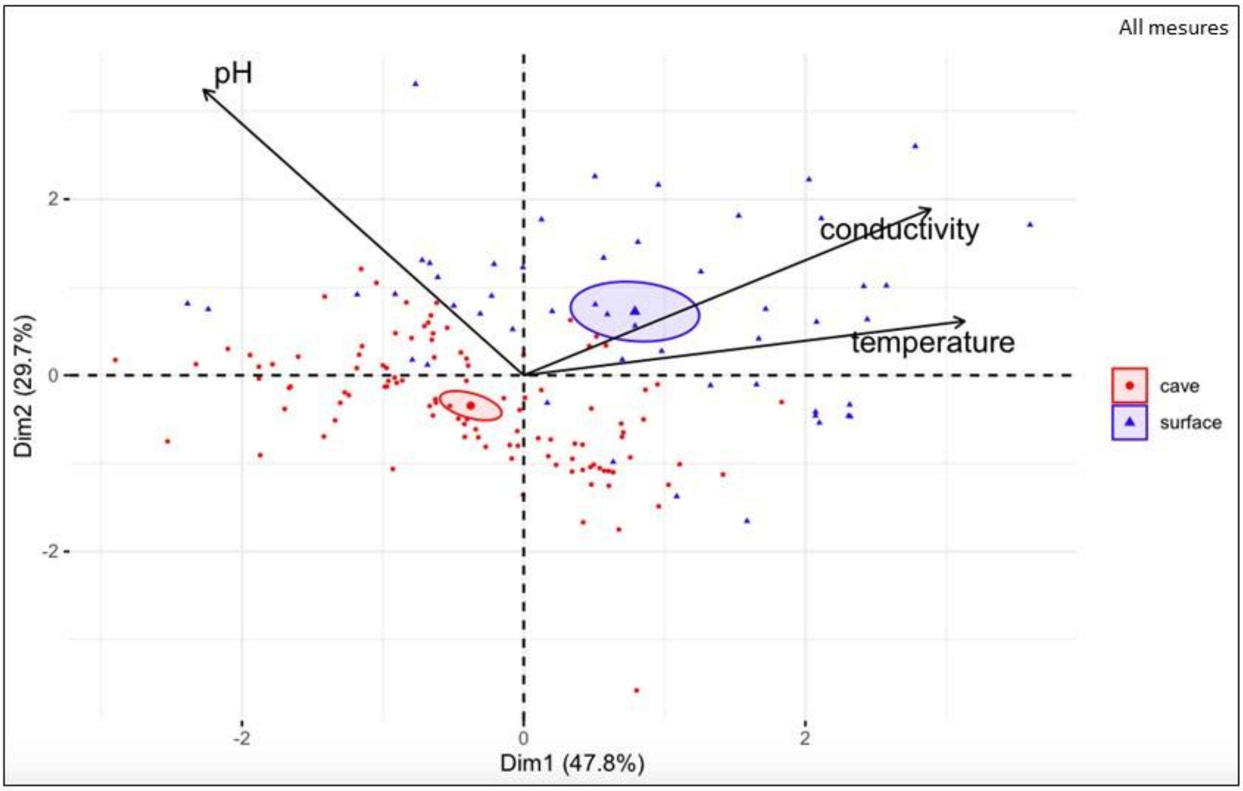

Supplemental Figure 2: Clustering of the physico-chemical properties of rivers and caves water using PCA.

This graph includes all values recorded with different instruments. Ncaves=107, Nsurface=51, N=3 variables. See **Supplemental File 2**, raw data. Color codes are indicated, 95% confidence ellipses are shown. Anova/pca1, pvalue=1.88e-09; Anova/pca2, pvalue=8.64e-13.

The results are identical to the analysis shown in **Fig. 2**, where only the values recorded from Combo Hanna are shown. This strongly suggests that measures recorded with different instruments are similar and therefore reliable in our dataset.

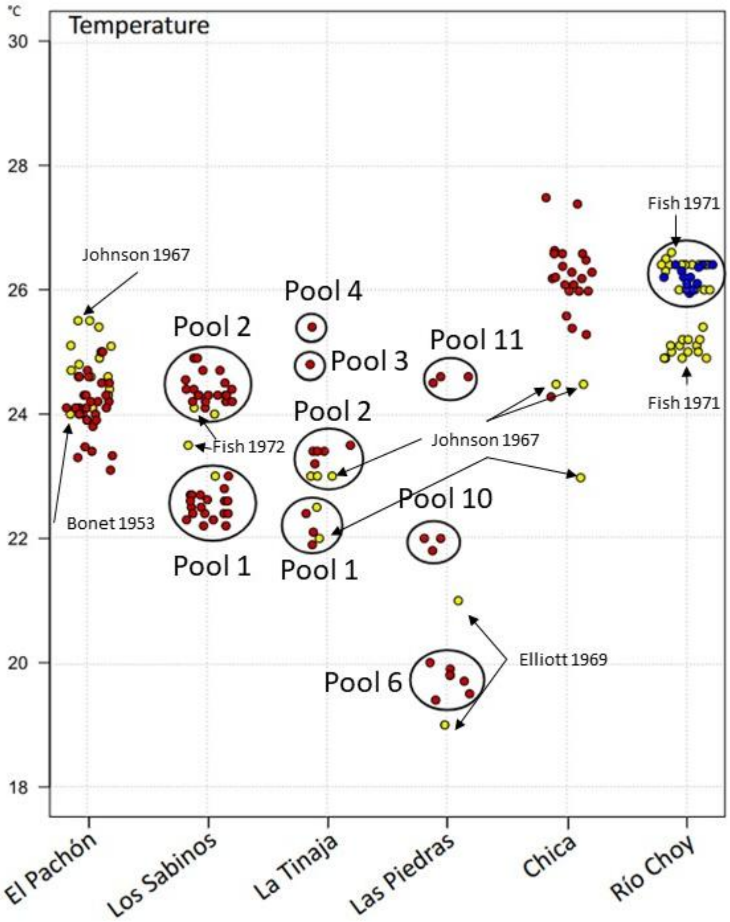

Supplemental Figure 3: Stability of water temperature measurements over 7 decades (1953-2024)

Same as Fig. 3, including also data collected for Cueva de Los Sabinos, Cueva de la Tinaja, Sótano de Las Piedras, Cueva Chica. Red dots (caves) and blue dots (river) are from our dataset. Black circles indicate cave pool numbers, known from our dataset. Yellow dots are from ancient literature, with the author name & date. However, in most cases there is no pool location information in these reports. Older measurements are in the range of ours, suggesting global stability of mean temperatures in these caves over 70 years.

For the surface station Nacimiento del Río Choy (blue dots; river location #3 on Fig.1A), a black circle shows a group of ours & old data (Fish, 2004) close to 26°C. Another group of only older data is close to 25°C, also from Fish (2004). Fish showed that temperature could vary within days with flooding (e.g., between 5 June and 14 August 1971) at this location, with also massive variations in discharge, turbidity and water chemistry.

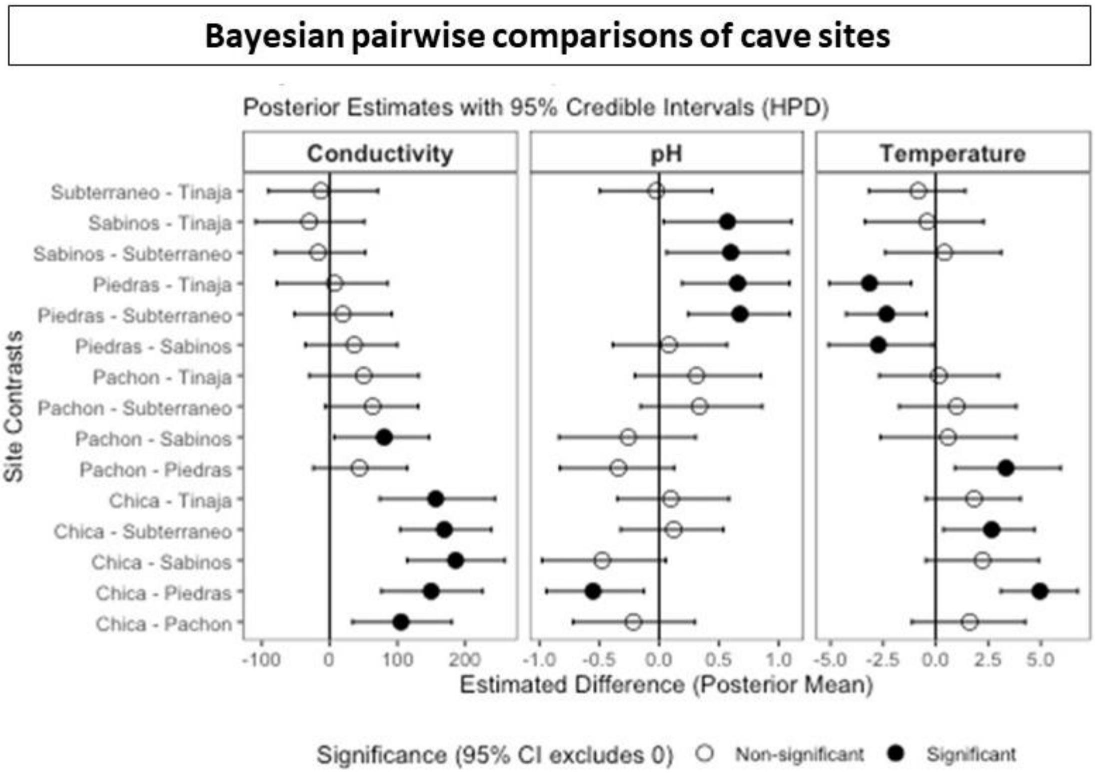

Supplemental Figure 4: Pairwise comparison of physico-chemical parameters of the water between caves.

Pairwise comparisons for the 6 caves studied (bmr, contrasts on emmeans, Tuckey's adjustement). Significant differences are represented by a filled circle (see Methods).

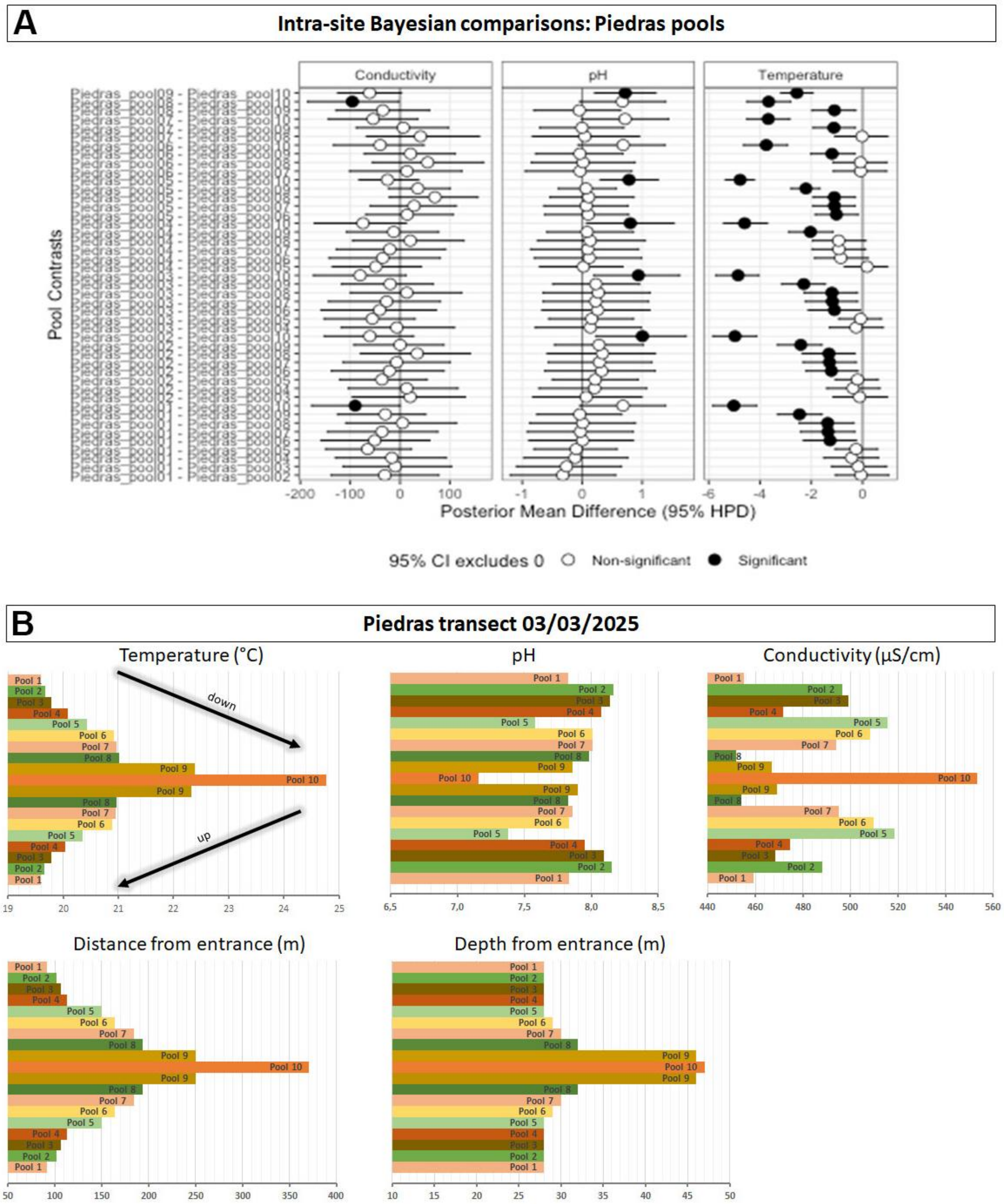

Supplemental Figure 5: Piedras transect study, pool1 to pool10, complete dataset.

A: Pairwise comparisons for the 10 pools in the Piedras cave (bmr, contrasts on emmeans, Tuckey's adjustement). Significant differences are represented by a filled circle.

B: Transect analysis in Piedras cave through pools 1 to 10, showing temperature, pH, conductivity, as well as distance from entrance and depth. The low-to-high temperature gradient from the first to last pool encountered seems to vary as a function of the distance from entrance, less so with depth of the pool. pH and conductivity do not show such a gradient.

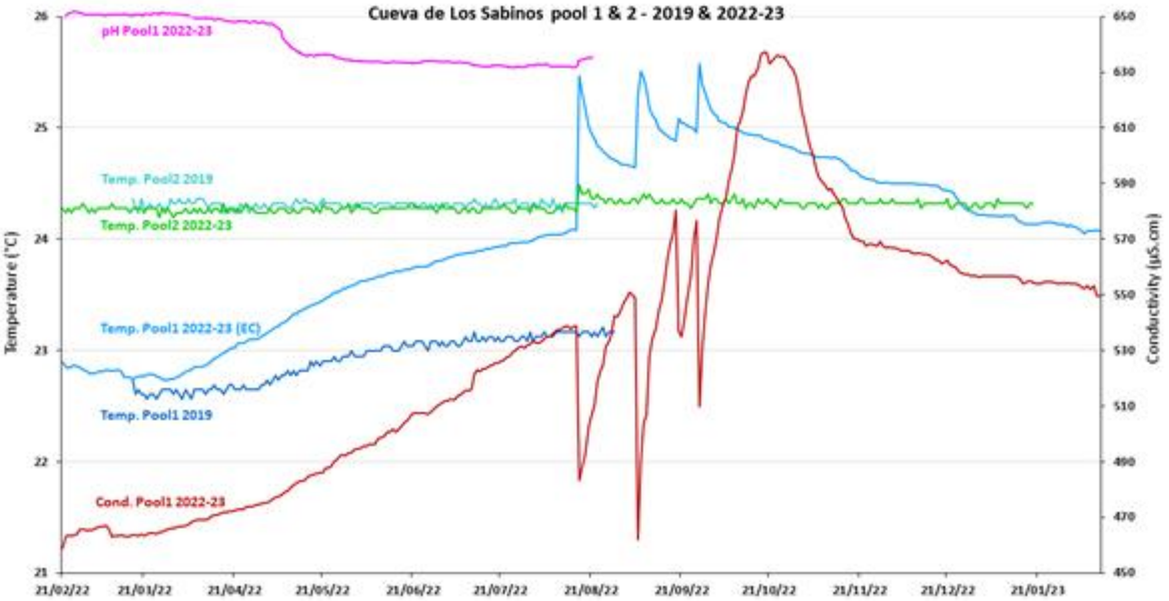

Supplemental Figure 6: Recordings in Sabinos

The evolution of temperature, pH and conductivity, throughout the year 2019 and from February 2022 to February 2023 in Sabinos cave pools 1 and 2. Color codes for recorded parameter, pool and year of interest are indicated. The temperature in Pool1, year 2022-23, was recorded in duplicate from the conductivity probe (EC, shown) and the pH probe (not shown, for graph clarity), and they showed exactly the same pattern with a slight shift of 0.3-0.4°C all along the curve. Note that Pool2 is globally warmer than Pool1 over two years except during the rainy season; that Pool1 seems to undergo more fluctuations than Pool2; and that different parameters vary in a correlated manner in Pool1 at a daily timescale.

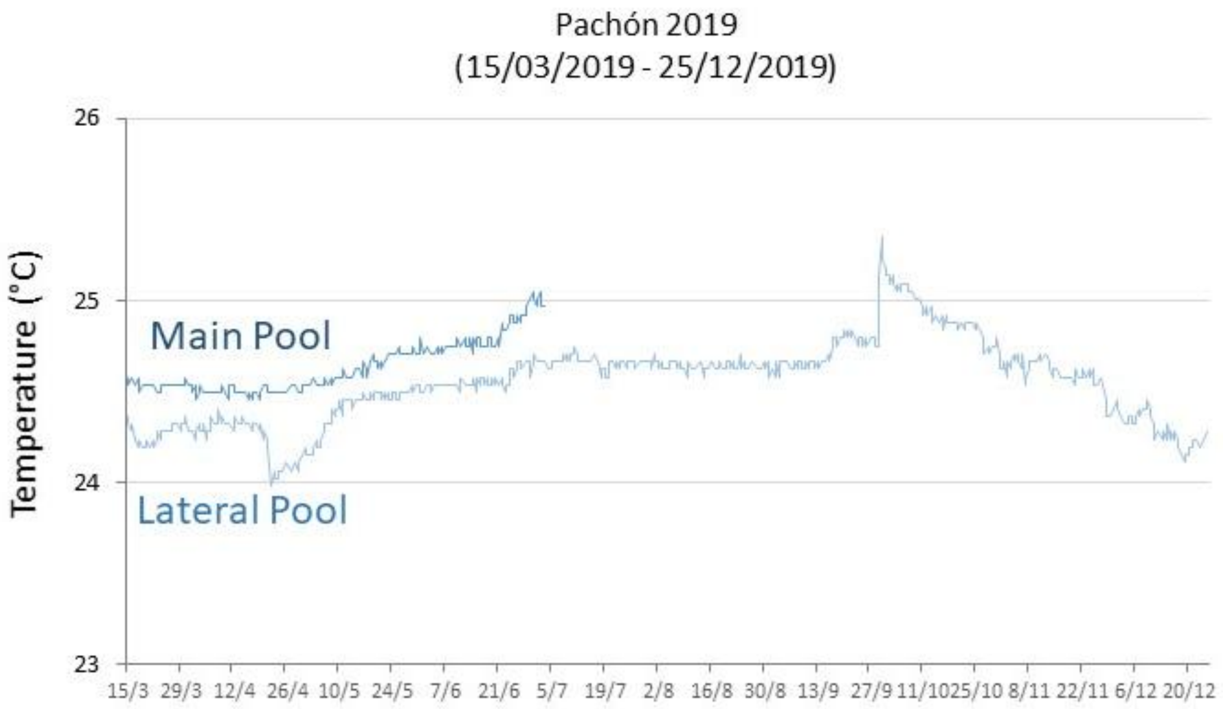

Supplemental Figure 7: Recordings of water temperature in Pachón in 2019

Evolution of temperature from 15 March 2019 to 25 December 2019 in the main and lateral pools of the Pachón cave (Hobo probes). Recordings lasted 4 months in the main pool and 9 months in the lateral pool. Contrarily to other caves, the temperature appears very stable, varying mostly in the range between 24°C and 25°C.
